# Supplementary material for: Analysis of GEN1 as a Breast Cancer Susceptibility Gene in Polish Women
Source: Int J Mol Sci. 2025 Jun 22;26(13):5991. doi: 10.3390/ijms26135991 (PMC12250413; doi:10.3390/ijms26135991)
Supplement: Supplementary file 1 [file ijms-26-05991-s001.zip › ijms-3660693-supplementary.pdf]

Supplementary Table S1. Geographical distribution of the GEN1 c.1929_1932delAAAG (p.Lys645Cysfs*29) deletion among 15,930 Polish women with unselected breast cancer, and among 4702 cancer-free female controls

	**Women with unselected breast cancer (n=15,930)**<br><br>Mean age: 56 years<br>Age range:18 – 94 years		**Cancer-free female controls (n=4702)**<br><br>Mean age: 53 years<br>Age range: 18 - 95 years	
**Region**	**City**	**Mutation frequency**	**City**	**Mutation frequency**
Northwestern Poland	Szczecin, Koszalin	0.24% (10/4234)	Szczecin, Koszalin	0.17% (4/2290)
Northeastern Poland	Olsztyn, Białystok	0.21% (6/2837)	Olsztyn, Białystok	0.25% (1/405)
Central Poland	Bydgoszcz, Toruń<br>Poznań, Warszawa<br>Łódź, Zielona Góra	0.27% (7/2641)	Bydgoszcz, Toruń<br>Poznań, Warszawa<br>Łódź, Zielona Góra	0.17% (1/582)
Southwestern Poland	Opole, Bielsko-Biała<br>Wrocław	0.26% (7/2706)	Opole, Bielsko-Biała<br>Świdnica	0.18% (2/1134)
Southeastern Poland	Kielce, Kraków<br>Lublin, Rzeszów<br>Brzozów	0.23% (8/3512)	Kielce, Kraków	0.00% (0/291)
All regions		0.24% (38/15,930)		0.17% (8/4702)

The adjusted Mantel–Haenszel odds ratio was calculated stratified by the geographical regions (the five geographical regions were considered as five separate studies). The adjusted OR was 1.34, 95% CI 0.62-2.87, p = 0.45, and Breslow-Day test of homogeneity of odds ratios was not significant (p = 0.65).
